# Ten simple rules for writing and sharing computational analyses in Jupyter Notebooks

**DOI:** 10.1371/journal.pcbi.1007007

**Published:** 2019-07-25

**Authors:** Adam Rule, Amanda Birmingham, Cristal Zuniga, Ilkay Altintas, Shih-Cheng Huang, Rob Knight, Niema Moshiri, Mai H. Nguyen, Sara Brin Rosenthal, Fernando Pérez, Peter W. Rose

**Affiliations:** 1 Design Lab, UC San Diego, La Jolla, California, United States of America; 2 Center for Computational Biology and Bioinformatics, UC San Diego, La Jolla, California, United States of America; 3 Department of Pediatrics, UC San Diego, La Jolla, California, United States of America; 4 Data Science Hub, San Diego Supercomputer Center, UC San Diego, La Jolla, California, United States of America; 5 Departments of Bioengineering, and Computer Science and Engineering, and Center for Microbiome Innovation, UC San Diego, La Jolla, California, United States of America; 6 Bioinformatics and Systems Biology Graduate Program, UC San Diego, La Jolla, California, United States of America; 7 Department of Statistics and Berkeley Institute for Data Science, UC Berkeley, and Lawrence Berkeley National Laboratory, Berkeley, California, United States of America; Whitehead Institute for Biomedical Research, UNITED STATES

## Introduction

As studies grow in scale and complexity, it has become increasingly difficult to provide clear descriptions and open access to the methods and data needed to understand and reproduce computational research. Numerous papers [1–3], including several in the Ten Simple Rules collection [4,5], have highlighted the need for robust and reproducible analyses in computational research, described the difficulty of achieving these standards, and enumerated best practices. We aim to augment this existing wellspring of advice by addressing the unique challenges and opportunities that arise when using computational notebooks, especially Jupyter Notebooks, for research [6].

Reproducibility, the scientific standard that others should be able to recreate your results, requires at a minimum that “data and the computer code used to analyze [that] data be made available to others” [2]. Achieving even this minimum standard typically requires both machine-readable descriptions of the data, software, dependencies, and computational environment involved (for example, hardware or cloud configuration), as well as human-readable documentation describing how all these pieces fit together. Whereas analysts previously kept code, documentation, and results in separate files, they increasingly use computational notebooks such as Jupyter Notebooks and R Notebooks to both perform analyses and combine code, results, and descriptive text in a single “computational narrative” to be read and rerun by others [7,8]. This ability to combine executable code and descriptive text in a single document has close ties to Knuth’s notion of “literate programming” [9] and has convinced many researchers to switch to computational notebooks from other programming environments. Jupyter Notebooks in particular have seen widespread adoption: as of December 2018, there were more than 3 million Jupyter Notebooks shared publicly on GitHub (https://www.github.com) [10], many of which document academic research [11].

The interactive and narrative nature of computational notebooks presents unique opportunities for performing and sharing computational research. With some forethought, they can provide not only richly detailed descriptions of analyses but also interactive computing environments for replicating, exploring, and extending them. Yet, as with other computing environments, using notebooks for research requires special care. Interactively running and editing code in notebooks can delete key steps or introduce “hidden state” that confounds analyses and confuses readers [12]. Analyses documented in notebooks cannot be easily rerun if users do not first freeze their dependencies, share their data, and adequately describe their computing environment [13]. And many notebooks lack sufficient descriptive text to guide readers in using them [11,14].

**Fig 1:**
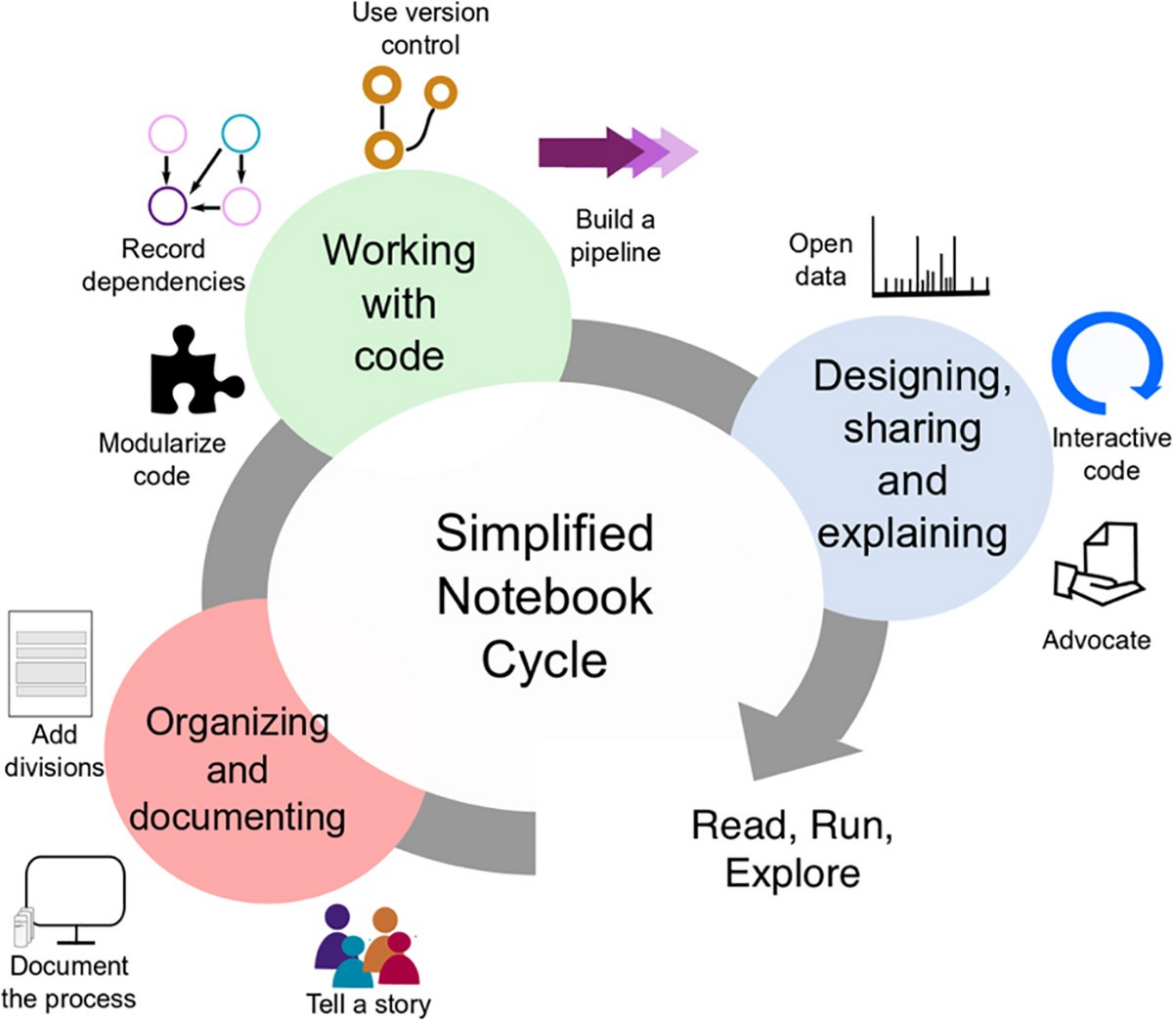
Iterative workflow for applying the 10 simple rules to the creation of Jupyter Notebooks. The cycle describes three overlapping phases of developing a well-documented and functional Jupyter Notebook. First, we organize and document the notebook (Rules 1–3). Second, the code is developed following the rules proposed here about quality standards (Rules 4–7). Finally, the notebook is made available, along with its data (Rule 8), in a manner encouraging public exploration and contribution (Rules 9–10).

The explosive growth of computational notebooks provides a unique opportunity to support computational research, but care must be taken when performing and sharing analyses in notebooks. Given these opportunities and challenges, we have compiled a set of rules, tips, tools, and example notebooks to help guide Jupyter Notebook authors. While we focus on a few core uses of Jupyter Notebooks observed in our own research, many of these rules can be applied to other computational notebooks and use cases. In Fig 1, we give a preview of the rules applied at different phases of the notebook development cycle. Whether you use notebooks to track preliminary analyses, to present polished results to collaborators, as finely tuned pipelines for recurring analyses, or for all of the above, following this advice will help you write and share analyses that are easier to read, run, and explore.

## Rule 1: Tell a story for an audience

One key benefit of using Jupyter Notebooks is being able to interleave explanatory text with code and results to create a computational narrative [7]. Rather than only keep sporadic notes, use explanatory text to tell a compelling story that has a beginning that introduces the topic, a middle that describes your steps, and an end that interprets the results. Describe not just what you did but why you did it, how the steps are connected, and what it all means. It is okay for your story to change over time, especially as your analysis evolves, but be sure to start documenting your thoughts and process as early as possible.

How you tell the story will depend on your goal and audience. Do you plan to share your notebook with a nontechnical colleague in your lab, analysts at another lab, readers of a particular journal, or the general public? You may need different kinds and levels of explanation for each audience. In any case, remember that your primary audience will most likely be your future self. Is your explanation clear enough that you will be able to understand and reproduce the analysis a month from now? People often overestimate what they will be able to remember in the future, so err on the side of overexplaining. If you won’t be able to recreate your own analysis in the near future, how could anyone else?

## Rule 2: Document the process, not just the results

Computational notebooks’ interactivity makes it quick and easy to try out and compare different approaches or parameters—so quick and easy that we often fail to document those interactive investigations at the time we perform them. Thus, the advice long provided regarding paper lab scientific notebooks becomes even more critical: make sure to document all your explorations, even (or perhaps especially) those that led to dead ends. These comments will help you remember what you did and why. You can always remove these comments later if turning your notebook into a pipeline (see Rule 7) or preparing to share it with a wider audience (Rule 1), who may prefer to see a concise presentation of results rather than a detailed lab notebook.

Many notebook users wait to add such explanatory text until the end of an analysis, after they have a solid result. Don’t wait—by that point you may have forgotten why you chose a particular parameter value, where you copied a block of code from, or what you found interesting about an intermediate result. If you do not have time to fully document what you are doing or thinking in the moment, leave short descriptive notes to remind yourself what to add when you get to a good stopping point. While the code needed to reproduce the analysis may be automatically captured in your notebook, the reasoning and intuition may not. It is okay if the story in your notebook changes over time; you should still tell a story from the very beginning, even if you don’t know the ending yet.

Clean, organize, and annotate your notebook after each experiment or meaningful chunk of work and do all your cleaning in the notebook. For example, when preparing to publish, avoid manually tweaking figures with desktop publishing tools and instead use plotting libraries with the notebook to produce publication-ready versions of figures and other artifacts to be used in manuscripts. Make sure you include your name as well as contact information for yourself and a future contact in your lab that can answer basic questions about the code. Documenting the beginning and end date of your analysis is also a good idea and can highlight the effort that you have put into the development of the notebook.

## Rule 3: Use cell divisions to make steps clear

Notebooks are an interactive environment, so it is very easy to write and run one-line cells. This supports experimentation but can leave your notebooks messy and full of short fragments that are hard to follow. Instead, try to make each cell in your notebook perform one meaningful step of the analysis that is easy to understand from the code in the cell or the surrounding markdown description. Modularize your code by cells and label the cells with markdown above the cell. Think of each cell as being one paragraph, having one function, or accomplishing one task (for example, create a plot).

Avoid long cells (we suggest that anything over 100 lines or one page is too long). Put low-level documentation in code comments. Use descriptive markdown headers to organize your notebook into sections that can be used to easily navigate the notebook and add a table of contents. Split long notebooks into a series of notebooks and keep a top-level index notebook with links to the individual notebooks. Using clear cell and notebook divisions will make your analysis much easier to read.

## Rule 4: Modularize code

It is always good practice to avoid duplicate code, but in notebooks, it is especially easy to copy a cell, tweak a few lines, paste the resulting code into a new cell or another notebook, and run it again. This form of experimentation is expedient but makes notebooks difficult to read and nearly impossible to maintain if you want to change the functionality of or fix a bug in the copied code. Instead, wrap code you are about to copy and reuse in a function, which you can then call from as many cells as desired. If you are going to reuse the code in other projects or notebooks, consider turning it into a module, package, or library.

Not only does modularization save space, support maintenance, and ease debugging, it also makes it easier to add interactivity. For example, you can tie widgets (ipywidgets, https://ipywidgets.readthedocs.io/en/stable/) to functions to support exploration of different parameter values or support interaction with visualizations without needing to modify the code. This is one way you can design your notebook to be explored (Rule 9).

## Rule 5: Record dependencies

Rerunning your analysis in the future will require accessing not only your code but also any module or library that your code relied on. As is best practice across computational science, manage your dependencies using a package or environment manager like pip or Conda. These enable you to download modules and libraries, specify the version of each you want to use in your analysis, and even generate files such as Conda’s environment.yml or pip’s requirements.txt that concisely describe all of your dependencies. These files can be used by tools such as Binder or Docker to generate a “container” that other researchers can use to reproduce your analysis using the same versions of every module and library as you did. Always conduct your work in an environment created only from these dependencies to ensure you do not add undocumented dependencies.

As an extra precaution in notebooks, you can explicitly print out your dependencies using a notebook extension such as watermark (https://github.com/rasbt/watermark). Listing the versions of critical dependencies in the notebook itself (best done at the bottom) will ensure that, if used in isolation from its environment, the notebook still contains critical information to help readers run it.

## Rule 6: Use version control

Version control is a critical adjunct to notebook use because the interactive nature of notebooks makes it easy to accidentally change or delete important content. Furthermore, since notebooks contain code and code inevitably contains bugs, being able to determine the history of when a given bug you have discovered was introduced to the code versus when it was fixed—and thus what analyses it may have affected—is a key capability in scientific computation. Consult the Ten Simple Rules paper by Perez-Riverol and colleagues [15] on how to take advantage of Git and GitHub for version control generally. Also follow best practices for organizing your repository for easy version control, for example, http://drivendata.github.io/cookiecutter-data-science/.

However, be aware that Jupyter Notebooks store both code and extensive metadata about each cell as a text file in the JavaScript Object Notation (JSON) format. Version control systems compare differences in these JSON files, not differences in the user-friendly notebook graphical user interface (GUI). Because of this, reported differences between versions of a given notebook are usually difficult for users to find and understand because they are expressed as changes in the abstruse JSON metadata for the notebook. One way to address this issue is to use a notebook-specific diffing tool like nbdime that understands notebook structure and presents differences in meaningful ways (https://github.com/jupyter/nbdime). Another approach is to convert your notebook to a more version-control–friendly filetype such as .py before committing changes.

## Rule 7: Build a pipeline

Notebooks documenting initial, exploratory investigations will rarely be widely generalizable, but once a stable analysis approach has been identified, a well-designed notebook can be generalized into a pipeline that easily repeats that analysis using different input data and parameters. With this end in mind, design your notebook from the beginning to allow such future repurposing. Place key variable declarations, especially those that will be changed when doing a new analysis, at the top of the notebook rather than burying them somewhere in the middle. Perform preparatory steps, like data cleaning, directly in the notebook and avoid manual interventions.

Because notebooks’ interactivity make them vulnerable to accidental overwriting or deletion of critical steps by the user, if your analysis runs quickly, make a habit of regularly restarting your kernel and rerunning all cells to make sure you did not accidentally delete a step while cleaning your notebook (and if you did, retrieve the code for it from version control). Restarting your kernel and running all cells is also a good final test of results. To allow partial execution of complex analyses, break long notebooks into smaller notebooks that focus on one or a few analysis steps. Then, ensure that each notebook stores serialized versions of key intermediate results to disk for subsequent notebooks to use.

Once a notebook has been developed, it can be parameterized with a tool such as papermill (https://github.com/nteract/papermill). Such notebooks can be used not only interactively but also as command-line tools that can be executed automatically—a great boon for pipelines! Consider linking your analysis pipeline steps via a Makefile or similar tool that allows for complete noninteractive execution of the entire pipeline, either in full or partial steps. Such automation also supports code quality techniques like software testing; consider testing your workflows from end to end each time a change is committed by integrating your repository to a Continuous Integration system (for example, https://travis-ci.org/). Last but not least, be aware that pipeline notebooks will almost certainly have a very different story (Rule 1) than the initial analyses that engendered them! Remember to remove any introduction, interpretation, or conclusion text that is not universally applicable to different inputs and results and instead replace it with guidance for the pipeline user on how to run and interpret its (potentially novel) results.

## Rule 8: Share and explain your data

Having access to a clearly annotated notebook is of little use to those wanting to reproduce or extend your results if the underlying data are locked away. Strive to make your data or a sample of your data publicly available along with the notebook. While sharing your data takes careful planning, notebooks make it easy to provide a description of your input data and upstream processing steps, which are essential for interpreting results.

Ideally, you will share your entire data set alongside your notebooks. We realize many data sets are too large or too sensitive to share this way. In these cases, consider breaking down large and complex data sets into tiers such that, even if the raw data are prohibitively large to include alongside your published notebooks or are constrained by privacy or other access issues, reproducibility and interpretability isn’t lost. You can host public copies of medium-sized, anonymized data in a variety of hosting services (for example, figshare [https://figshare.com/], zenodo [https://zenodo.org/]), and include further processed data sets alongside the notebooks in the final repository. To uniquely and permanently identify data sets, these hosting services provide Digital Object Identifiers (dois). This tiered approach both provides public confidence and allows others to reproduce and reuse the latter stages of an analysis even without access to the full, raw data set.

## Rule 9: Design your notebooks to be read, run, and explored

If you have followed the previous rules, your notebooks should capture your entire process and be easy to read. But how will others access, run, and explore them? There are a number of ways you can support others’ reuse of your notebooks. First, store your notebooks in a public code repository with a clear README file and a liberal open source license (https://opensource.org/licenses) granting permission to reuse your code.

Read: Beyond granting permission to reuse your notebook, consider how you can leverage the unique structure of notebooks to support reading. At the very least, leave static HTML/PDF versions of all notebooks stored in the final version of the repository accompanying a publication. If, in 20 years, all other execution technology fails, these are likely to still provide a readable archival record, and with a full dependences list, future users are more likely to be able to recreate the compute environment. You can also use Nbviewer (https://nbviewer.jupyter.org/) to provide static views of your executed notebook online without needing to convert it to a PDF/HTML document first. GitHub uses this service to render any notebooks on their site, so pushing a notebook to GitHub is another good way to make static views easily available. In both cases, you can point collaborators to a URL where they can read through your notebook online.

Run: To support others running your notebooks, you can use Binder [16] to provide a zero-install environment to run your notebooks in the cloud (https://mybinder.org/). Binder enables community members to rerun your notebook online without needing to install Jupyter Notebook or Jupyter Lab on their own machine. More generally, you can create a portable containerized environment, for example, a Docker image (https://docs.docker.com/), or create a dependency description file (see Rule 3) so future users of your notebook can more easily replicate your computing environment when rerunning your notebook.

Explore: Beyond simply replicating the analysis in your notebook, consider how you can design your notebook so future users can tweak and explore your analysis. Consider using ipywidgets (https://ipywidgets.readthedocs.io/en/stable/) to enable future users to change parameters using graphical elements such as dropdowns and sliders rather than tweaking code. Beyond enabling future users to change parameters or insert their own data set, consider how they might want remix or reuse portions of your notebook (perhaps only the data cleaning or plotting steps) and use cell-structure and functions to make it easier to extract these sections (Rule 7).

## Rule 10: Advocate for open research

Clearly, the mere use of a computational notebook does not guarantee others will be able to read, run, or explore your analysis. If the convenience and interactivity of this technology has convinced you to adopt it, take the next step and become an advocate in your lab or workplace in promoting its effective use. Ask lab-mates or colleagues to try to run one of your notebooks and then listen when they explain any difficulties. Try to run their notebooks and let them know if you hit snags. Commit yourself to robust and reproducible analyses as key element of all your research group’s computational work, not a phase performed after an analysis is complete or an afterthought triggered by journal or reviewer demands.

## Annotated notebooks

To demonstrate the 10 rules, we have created a Git Repository with annotated example notebooks (https://github.com/jupyter-guide/ten-rules-jupyter). Following Rule 9, read, run, and explore these notebooks. In addition, we have created a repository (https://github.com/jupyter-guide/jupyter-guide) to crowdsource more technical and in-depth tutorials and to keep up with the rapidly evolving Jupyter ecosystem. We encourage you to contribute and share your experiences and know-how following Rule 10.

## Conclusions

Robust and reproducible analyses lie at the heart of science, and several papers have already provided excellent general advice for how to perform and document computational science. However, the advent of computational notebooks presents new opportunities and challenges, both easing precise documentation of complex workflows, and complicating it by means of interactivity. We present 10 simple rules for writing and sharing analyses in Jupyter Notebooks, focusing on annotation of the analysis, organization of code, and ease of access and reuse. Informed by our experience, we hope they contribute to the ecosystem of individuals, labs, publishers, and organizations using notebooks to perform and share computational research.

## References

[1] Barba LA. The hard road to reproducibility. Science. 2016;354: 142 10.1126/science.354.6308.142 27846503

[2] Peng RD. Reproducible Research in Computational Science. Science. 2011;334: 1226–1227. 10.1126/science.1213847 22144613 PMC3383002

[3] Wilson G, Bryan J, Cranston K, Kitzes J, Nederbragt L, Teal TK. Good enough practices in scientific computing. PLoS Comput Biol. 2017;13(6):e1005510 10.1371/journal.pcbi.1005510 28640806 PMC5480810

[4] Sandve GK, Nekrutenko A, Taylor J, Hovig E. Ten simple rules for reproducible computational research. PLoS Comput Biol. 2013;9(10):e1003285 10.1371/journal.pcbi.1003285 24204232 PMC3812051

[5] Taschuk M and Wilson G. Ten simple rules for making research software more robust. PLoS Comput. Biol. 2017;13(4):e1005412 10.1371/journal.pcbi.1005412 28407023 PMC5390961

[6] Reproducible Research using Jupyter Notebooks. [Internet] [cited 4 Oct 2018]. Available from: https://reproducible-science-curriculum.github.io/workshop-RR-Jupyter/.

[7] Pérez F, Granger BE. Computational Narratives as the Engine of Collaborative Data Science. 2015. [Internet] [cited 4 Oct 2018]. Available from: https://blog.jupyter.org/project-jupyter-computational-narratives-as-the-engine-of-collaborative-data-science-2b5fb94c3c58.

[8] Kluyver T, Ragan-Kelley B, Pérez F, Granger B, Bussonnier M, et al Jupyter Notebooks—a publishing format for reproducible computational workflows In: Loizides F, Schmidt B, editors. Positioning and Power in Academic Publishing: Players, Agents and Agendas. Amsterdam: IOS Press; 2016 p. 87–90. 10.3233/978-1-61499-649-1-87

[9] Knuth DE. Literate programming. The Computer Journal. 1984;27(2):97–111.

[10] Estimate of Public Jupyter Notebooks on GitHub. [Internet] [cited 4 Oct 2018]. Available from: https://github.com/parente/nbestimate.

[11] Rule A, Tabard A, Hollan JD. Exploration and Explanation in Computational Notebooks. CHI '18 Proceedings of the 2018 CHI Conference on Human Factors in Computing Systems. New York: ACM; 2018. 10.1145/3173574.3173606

[12] Grus, J. I Don’t Like Notebooks. JupyterCon. New York, NY. 2018. [Internet] [cited 3 Jan 2019]. Available from: https://docs.google.com/presentation/d/1n2RlMdmv1p25Xy5thJUhkKGvjtV-dkAIsUXP-AL4ffI/edit#slide=id.g3d168d2fd3_0_255

[13] Woodbridge M, Sanz D, Mietchen D, Mounce R. Jupyter Notebooks and reproducible data science. 2017 [Internet] [cited 4 Oct 2018]. Available from: https://markwoodbridge.com/2017/03/05/jupyter-reproducible-science.html.

[14] Kery MB, Radensky M, Arya M, John BE, Myers BA. The Story in the Notebook: Exploratory Data Science using a Literate Programming Tool. CHI '18 Proceedings of the 2018 CHI Conference on Human Factors in Computing Systems. New York: ACM; 2018. 10.1145/3173574.3173748

[15] Perez-Riverol Y, Gatto L, Wang R, Sachsenberg T, Uszkoreit J, et al Ten Simple Rules for Taking Advantage of Git and GitHub. PLoS Comput. Biol. 2016;12(7):e1004947 10.1371/journal.pcbi.1004947 27415786 PMC4945047

[16] Project Jupyter, Bussonnier M, Forde J, Freeman J, Granger B, et al. Binder 2.0—Reproducible, interactive, shareable environments for science at scale. Proceedings of the 17th Python in Science Conference 2018. 2018. p. 113–120. 10.25080/Majora-4af1f417-011

